# The predator problem and PCR primers in molecular dietary analysis: Swamped or silenced; depth or breadth?

**DOI:** 10.1111/1755-0998.13705

**Published:** 2022-09-10

**Authors:** Jordan P. Cuff, James J. N. Kitson, David Hemprich‐Bennett, Maximillian P. T. G. Tercel, Samuel S. Browett, Darren M. Evans

**Affiliations:** ^1^ School of Natural and Environmental Sciences Newcastle University Newcastle upon Tyne UK; ^2^ Department of Zoology University of Oxford Oxford UK; ^3^ School of Biosciences Cardiff University Cardiff UK; ^4^ Durrell Wildlife Conservation Trust, Les Augrès Manor, La Profonde Rue Trinity Jersey; ^5^ Ecosystems and Environment Research Centre, School of Science, Engineering and Environment University of Salford Salford UK

**Keywords:** amplification, DNA metabarcoding, food webs, molecular analysis, trophic interactions

## Abstract

Dietary metabarcoding has vastly improved our ability to analyse the diets of animals, but it is hampered by a plethora of technical limitations including potentially reduced data output due to the disproportionate amplification of the DNA of the focal predator, here termed “the predator problem”. We review the various methods commonly used to overcome this problem, from deeper sequencing to exclusion of predator DNA during PCR, and how they may interfere with increasingly common multipredator‐taxon studies. We suggest that multiprimer approaches with an emphasis on achieving both depth and breadth of prey detections may overcome the issue to some extent, although multitaxon studies require further consideration, as highlighted by an empirical example. We also review several alternative methods for reducing the prevalence of predator DNA that are conceptually promising but require additional empirical examination. The predator problem is a key constraint on molecular dietary analyses but, through this synthesis, we hope to guide researchers in overcoming this in an effective and pragmatic way.

## 1 DNA METABARCODING, DIETARY ANALYSIS AND THE PREDATOR PROBLEM

Accurate knowledge of trophic interactions is crucial for understanding everything from the behaviour of individuals to ecosystem processes and how they respond to change (Thébault & Loreau, 2005). Traditional methods for dietary analysis, such as analysis of hard parts, overlook or fail to identify soft‐bodied or morphologically cryptic prey (Jeanniard‐du‐Dot et al., 2017; Nielsen et al., 2018). By facilitating the accurate identification of prey from minute and degraded remains, molecular methods, such as DNA metabarcoding, have significantly advanced our ability to analyse the diets of animals in nature (Jeanniard‐du‐Dot et al., 2017; Pompanon et al., 2012; Symondson, 2002). There are, however, many experimental biases inherent to metabarcoding, the intrinsic biases of PCR amplification being among the most pervasive. These biases compound an innate problem of predatory dietary analysis: that of the high prevalence of predator DNA detected in dietary samples.

In most cases, dietary metabarcoding studies aim to identify the dietary composition of a consumer as comprehensively as possible, but the taxonomic biases of PCR primers confound this. Mismatches between primers and target DNA result in inefficient annealing, causing amplification bias or prevention of amplification entirely (Piñol et al., 2018). Such biases cause a nonlinear relationship between starting and amplified concentrations of DNA of each species detected (Paula et al., 2015), inhibiting accurate quantification of input DNA concentrations from sequencing outputs (Piñol et al., 2018; Stadhouders et al., 2010). Metabarcoding PCR primers for dietary analysis of a predator ideally amplify the DNA of the full range of potential prey species contained in that predator's gut contents or faeces, but all primers ultimately fail to amplify some taxa (Brandon‐Mong et al., 2015; Elbrecht & Leese, 2017; Mao et al., 2012), so compromises must often be made. These biases are particularly problematic in dietary studies concerning generalist predators which exploit taxonomically diverse resources. Richer samples increase the likelihood of problematic bias by demanding a greater range of detections from the same read depth, and phylogenetically diverse samples are more likely to fall victim to the taxonomic biases of PCR due to a higher likelihood of mismatches with the primer (Deagle et al., 2019). To further complicate matters, the diverse diets of generalists can often include taxa closely related to the predator itself, or even conspecific prey indistinguishable from the focal predator. This often results in the use of PCR primers which amplify the already highly prevalent DNA of the predator itself, reducing the data output attributed to prey.

The amplification of consumer DNA is most relevant to dietary analyses of predatory animals, and specifically in studies for which the PCR primers used amplify a sufficiently broad taxonomic range, consequently amplifying DNA of both the predator and its prey (e.g., reptiles feeding on both vertebrates and invertebrates; Tercel et al., 2022; bats feeding on a broad range of insects; Tournayre et al., 2020; spiders predating invertebrates including other spiders; Cuff, Tercel, et al., 2022). Other instances may be affected by this problem, such as metabarcoding‐based assessment of parasitism (Miller et al., 2021), filter‐feeding (Siegenthaler et al., 2018) or use of invertebrate‐aggregated DNA for biodiversity surveys (“iDNA”; Cutajar & Rowley, 2020; Drinkwater et al., 2021). Herbivorous dietary analyses will mostly circumvent this issue, although they are subject to their own limitations, particularly when concerning omnivores (Tercel et al., 2021). The “predator problem” is both quantitative and qualitative; that is, the predator's own DNA will often outnumber that of the prey, but will also be intact and undigested. Amplification of the dilute and digested prey DNA will be much less efficient than that of the predator DNA, thus it is likely to dominate the PCR product (Paula et al., 2015; Vestheim & Jarman, 2008; Waldner et al., 2013), leading to predator DNA comprising up to or over 95% of the sequencing output (Cuff et al., 2021; Piñol et al., 2014). Not only does this restrict the value of the data output of dietary studies by reducing the proportion of it attributed to prey, but it restricts the ability of researchers to easily identify and remove contamination (which can be common when using such sensitive methods) given that prey DNA may be present in such low proportions of samples (Drake et al., 2022), and it impacts the already questionable potential for quantification of sequencing outputs due to the reduced accuracy of read counts when they are significantly smaller values.

**FIGURE 1:**
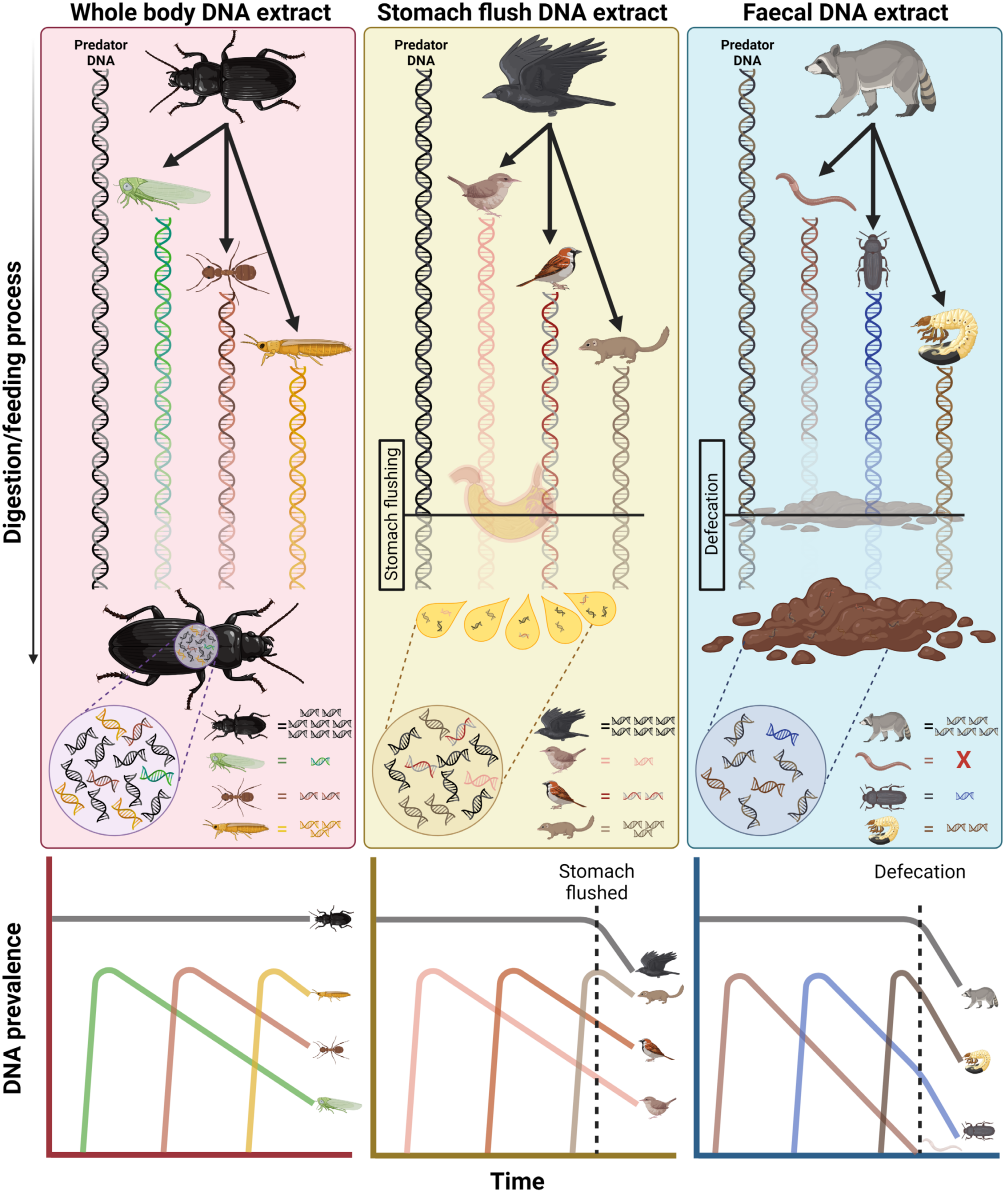
The hypothetical degradation of DNA throughout the digestive process preluding whole body DNA extraction (left), stomach flush or regurgitate DNA extraction (centre), and faecal DNA extraction (right). Line graphs below illustrate DNA prevalence over time. In whole bodies, prey DNA degrades linearly, but predator DNA remains constantly prevalent. In regurgitates, DNA prevalence will decrease following removal from the predator's body and will then degrade alongside the prey DNA. In faeces, predator DNA will be less prevalent following defecation, and will then degrade with prey DNA, which will have degraded throughout the entire digestive process. Figure created in Biorender.com

There are some circumstances that may modify these relationships, notably different sample types or predator physiologies (Figure 1). The predator DNA present in faecal samples, often used in dietary metabarcoding (Kaunisto et al., 2017; Pompanon et al., 2012; Rytkönen et al., 2019; Tercel et al., 2022), may be more degraded than that of gut contents of whole‐body extracts, but prey DNA is also likely to be more degraded. Regurgitates, commonly extracted from birds voluntarily (Ravache et al., 2020) or via stomach flushing, but also taken even from some invertebrates like carabid beetles (Kamenova et al., 2018), are commonly used for molecular dietary analysis and theoretically contain a far smaller proportion of predator DNA than in whole‐body extracts (e.g., for invertebrates with diverticulating guts; Macıas‐Hernández et al., 2018) and generally fresher prey DNA than that found in faeces. The feeding mode of predators is also important to consider; for example, arachnids tend to digest their prey externally, lending to greater degradation of prey DNA even before it enters the predator's gut. The same would also be the case for scavengers that feed on decaying animals (Calder et al., 2005; King et al., 2008). Such variables must be considered in each case when predicting how debilitating the predator problem is likely to be.

Predator DNA data can be useful for reliably identifying cryptic or difficult‐to‐identify predator species (Cuff et al., 2021; Tournayre et al., 2020, 2021), or, if the metabarcoding amplicon is sufficiently informative, to assess relatedness within populations and integrate ecoevolutionary context in downstream analyses (Derocles et al., 2018; Handley et al., 2011), but such instances are rare. Some of the DNA attributed to the focal predator may also belong to cannibalized conspecifics, possibly increasing the perceived prevalence of problematic predator DNA (i.e., increasing the predator problem). While cannibalism can be ecologically and nutritionally significant, the distinction between cannibalism and predator detection is impossible with most commonly used short metabarcoding markers due to insufficient intraspecific sequence variation, resulting in the treatment of these detections as focal predator DNA. Despite some potential utility, detection of focal predator DNA is most often problematic, consuming large amounts of the potential sequencing output which would otherwise be descriptive of the diet of the predators assessed. This problem is also pertinent to other metabarcoding applications such as parasitism (Miller et al., 2021). Here we discuss the dietary metabarcoding predator problem in depth, how this problem may be exacerbated in increasingly common multitaxon studies, but also several solutions, both common and novel.

## 2 COMPARING COMMON STRATEGIES FOR OVERCOMING THE PREDATOR PROBLEM

There are many solutions to the problem of predator amplification in dietary metabarcoding, each with its own set of pitfalls. First, the most conceptually straightforward approach is to use PCR primers that amplify a broad range of taxa but also knowingly amplify predator DNA. In such cases, the sequencing output can be dominated by predator reads, leaving little data for analysis of the prey consumed, but the taxonomic breadth of the prey detected is less restricted (Cuff et al., 2021; Piñol et al., 2014). This is a viable approach to dietary analysis so long as sufficient sequencing depth is achieved to identify a great enough proportion of the prey for the study's aims, but this increases the costs associated with the analysis and imposes additional technological constraints (i.e., higher capacity sequencers are necessary). Careful selection of tissues for optimal prey DNA presence can mitigate this issue to some degree (e.g., in spiders, the greatest prevalence of prey DNA existis in the abdomen, but the cephalothorax and even the femurs can contain viable concentrations of prey DNA; Macıas‐Hernández et al., 2018). This is not, however, possible for all predators and the proportion of predator DNA is usually an unpredictable and pervasive problem nonetheless. Importantly too, even the most general primers will exhibit some degree of bias and may fail to amplify some taxa (Brandon‐Mong et al., 2015; Elbrecht & Leese, 2017; Mao et al., 2012).

**FIGURE 2:**
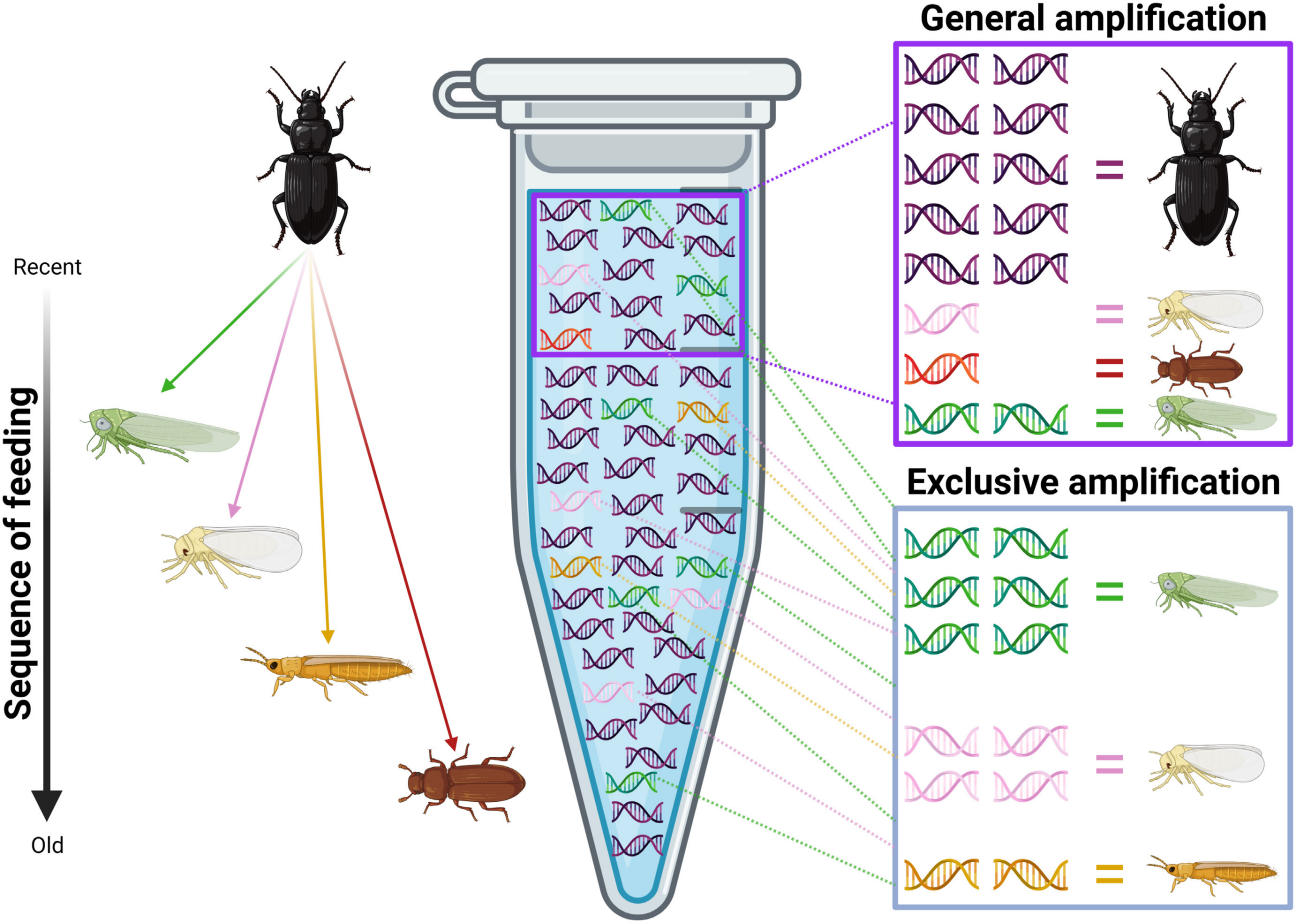
General PCR primers will amplify a broad taxonomic range, but most of the PCR product will be the predator itself, resulting in some species being omitted in the sequencing output. Exclusion primers will avoid amplification of the predator, but also some prey groups, particularly those phylogenetically close to the predator (e.g., the flour beetle being eaten by the carabid beetle in this image). Figure created in Biorender.com

Second, primers can also be designed carefully with a comprehensive reference database to amplify only target taxa, excluding (or at least exhibiting bias against) the predator's DNA (Ammann et al., 2020; Lafage et al., 2019; Zeale et al., 2011; Figure 2). Such “exclusion primers” can serendipitously occur among existing primers, for example those designed by Zeale et al. (2011) which have demonstrated exclusion of many taxa other than the bats they were designed to exclude (Berman & Inbar, 2021; Mitchell et al., 2021). These primers can, however, have very specific biases that might not apply, or may apply unevenly, to even confamilial taxa (Cuff et al., 2021), reducing their utility across studies or for multitaxon studies (the latter discussed in greater depth later). This exclusion may also extend far beyond the focal predator and could disrupt amplification of potential prey, especially if those prey are phylogenetically proximate to the focal predator, as can often be the case in intraguild predation (Cuff, Tercel, et al., 2022; Hambäck et al., 2022).

Third, blocking probes are a similar solution to exclusion primers, but are separate oligonucleotides that physically prevent amplification of a given taxon by preventing the PCR primers from annealing to primer sites (Deagle et al., 2009; Vestheim & Jarman, 2008). These prevent amplification of the DNA of specific taxa but, similarly to exclusion primers, can introduce biases of their own by incidentally blocking amplification of prey taxon DNA with similar priming site sequences (Murray et al., 2011; Piñol et al., 2015, 2018). Since blocking primers can be used alongside general primers, they can facilitate a taxonomically broad dietary analysis whilst specifically excluding predators (e.g., for pigs, Robeson et al., 2018; otters, Pertoldi et al., 2021; wolves and coyotes, Shi et al., 2021), but their direct competition with PCR primers and the exclusion of prey phylogenetically proximate to the predator can result in greater stochasticity of their success (Piñol et al., 2015). Successful application of blocking primers also requires accurate identification of the predator prior to molecular analysis; this is not always possible, especially for cryptic species, juveniles of many taxa, indirectly collected samples (e.g., faeces) and specimens for which morphological details are obscured, for example if animals are damaged during collection or storage (Cuff et al., 2021; Tournayre et al., 2020, 2021).

The choice of either attempting to silence or potentially being swamped by predator DNA is an insidious one given the financial, practical and experimental implications. The increased adoption of multiprimer (often multimarker, i.e., across multiple genes) metabarcoding (e.g., Batuecas et al., 2022; Cuff, Tercel, et al., 2022; da Silva et al., 2019, 2020; Stenhouse et al., 2021; Tercel et al., 2022) offers the option of using both approaches in synergy, but is by no means a panacea. Multimarker metabarcoding has been more generally suggested as a means of overcoming the issues associated with PCR primer bias (Browett et al., 2021; Cuff, Windsor, et al., 2022; da Silva et al., 2019). Given that each PCR primer pair exhibits a distinct taxonomic bias which profoundly impacts data output (Alberdi et al., 2018; Taberlet et al., 2018), the use of multiple markers can sometimes balance out the biases of each individually by generating distinct but overlapping data sets (da Silva et al., 2019). This approach is also advantageous for investigating taxonomically distinct food groups, such as vertebrates and arthropods (Drake et al., 2022) or, increasingly, plants and animals (Tercel et al., 2021), although such compartmentalisation is separate to the alleviation of bias given that these compartments will be subject to their own biases with little to no corrective overlap between them. Commitment to a combination of distinct primer pairs does not, however, answer the critical question of which primers to use and how best to tackle the predator problem, even if using multiple approaches (for comparisons of metabarcoding primers see Browett et al., 2021; Elbrecht et al., 2019; Piñol et al., 2018; Tournayre et al., 2020).

## 3 THE PREDATOR PROBLEM IN MULTITAXON DIETARY STUDIES: DEPTH OR BREADTH

The predator problem is compounded, regardless of approach(es), in studies concerning multiple predator taxa. If using general primers, the bias exhibited toward the predator may vary between the different predator taxa, resulting in uneven read depth attributed to those different predators. General primers are, however, by their nature less biased than the alternatives (Krehenwinkel, Wolf, et al., 2017) and this problem is only deepened when using exclusion or blocking primers. In these latter cases, a fundamental decision must be made: either separately use a different exclusion/blocking primer for each predator taxon, in which case the independent taxonomic biases of each will probably provide taxonomically distinct prey detections, confounding direct comparison, or use a single exclusion/blocking primer universally across all samples, in which case some predator taxa may lose more data output than others to the predator problem. This is ultimately a question of compromising depth (i.e., read depth evenness) or breadth (i.e., taxonomic breadth evenness), the answer to which may depend on the context of the specific study or research questions. It is possible to address both by amplifying every sample with a full series of exclusion primers, each suiting a different predator, but this quickly multiplies costs and labour. Equally, a “cocktail” of blocking/exclusion primers could be multiplexed in a single reaction, but they may compete, resulting in unpredictable permutations of their individual biases, and with more primers it becomes increasingly difficult to optimize PCR conditions for all of them in one reaction.

**FIGURE 3:**
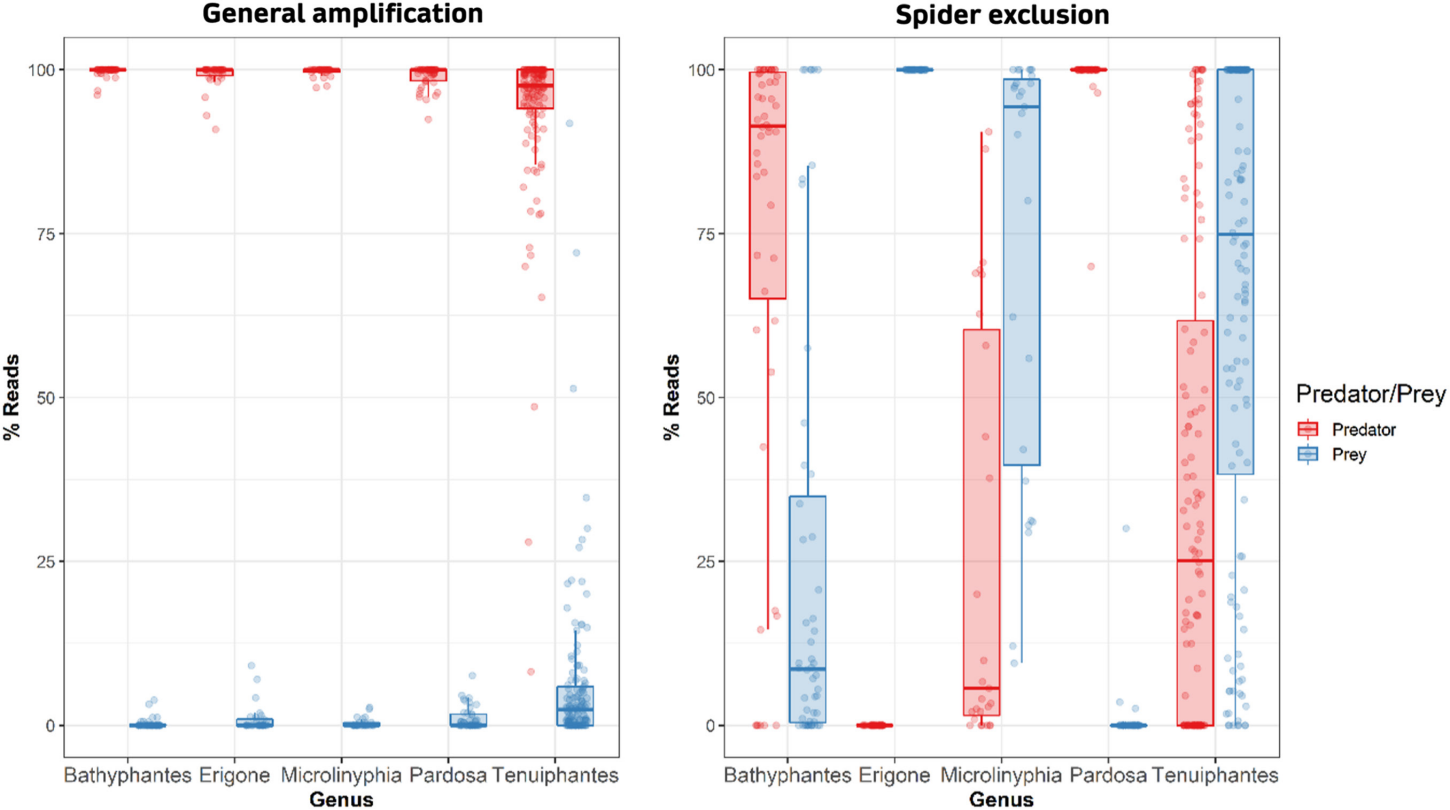
The percentage of reads attributed to predator and prey for each spider genus across two primer pairs used by Cuff, Tercel, et al. (2022). Colours distinguish between predator (red) and prey (blue) reads, and read counts are given as percentages of the total read count per sample. Figure originally presented by Cuff, Tercel, et al. (2022)

Cuff, Tercel, et al. (2022) presented a figure displaying the proportion of predator and prey reads in a dietary analysis of five spider genera using both general and exclusion primers (Figure 3). Whilst the general primers showed overall consistency in that the predator typically comprised 95%–100% of the sequencing output across all genera, the exclusion primers were highly inconsistent. For *Erigone*, no predator DNA was recovered, whilst for the other Linyphiidae genera, exclusion was inconsistent (predator DNA comprised mean 43.86% ± 39.86% of reads, ranging from 0% to 100%); at the other extreme, for the Lycosidae genus *Pardosa*, the vast majority of reads were lost to the predator (mean 99.14% ± 4.60% of reads, ranging from 69.98% to 100%; Table S1). The primers used were initially designed for the exclusion of linyphiids and were subsequently optimized for this purpose (Cuff et al., 2021), but they were used in this study even for the lycosid genus *Pardosa* to favour consistency of prey amplification despite lycosid exclusion primers existing (Lafage et al., 2019). The sequencing depth for each sample in this study was relatively large (including predator reads, 24,104 mean reads per sample for the general primers, and 15,004 mean reads per sample for the exclusion primers), still affording a relatively high and even detection of prey across samples (not accounting for the number of reads attributed to each prey taxon), but the results are indicative of a wider problem in dietary metabarcoding, especially as we transition toward increasingly multitaxon studies.

**FIGURE 4:**
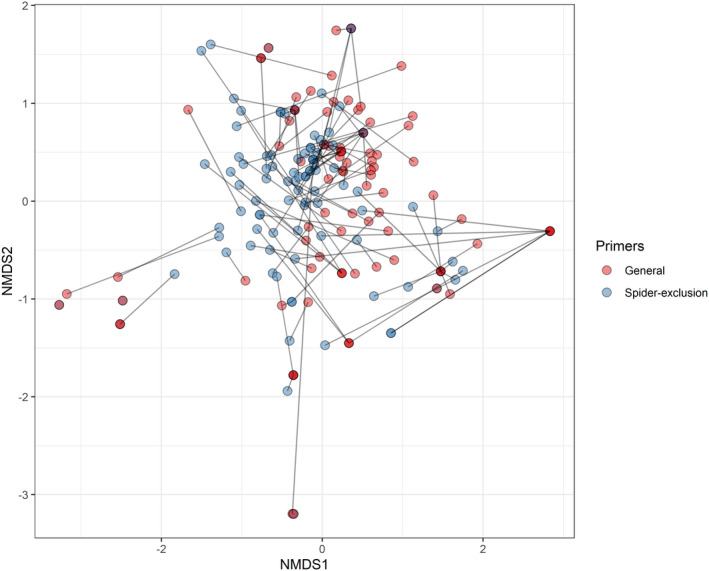
Nonmetric multidimensional scaling showing higher similarity of dietary data from the same primer pairs (colours; red and blue denoting general and exclusion primers, respectively) than within samples (points linked by black lines belong to the same sample). Creation of this figure is described in Appendix S1

The use of general and exclusion primers together for the same dietary samples provided a far greater diversity of prey than would be detected using just one. That both primers exhibited taxonomic biases is unsurprising, but their complementary biases highlight the strength of this approach. In fact, greater similarity can generally be observed within primer pairs than within samples, with the prey detected by each primer pair generally presenting overlapping but unique assemblages due to these biases (Figure 4). If different exclusion primers were used for each predator, this may have resulted in similarly distinct prey detections for each, artificially inflating the differences in their diets. By using a consistent exclusion primer, the loss is restricted to the sequencing depth but, since this was sufficient to detect a high diversity of prey even with the general primers, this is theoretically not debilitating. An additional option would be the inclusion of another exclusion primer suitable for lycosids such as NoSpi (Lafage et al., 2019) alongside the existing two primer pairs, but applied across all samples. Unless they were multiplexed as discussed above, this approach would further multiply the costs of metabarcoding, but would be a viable means for reducing the uneven loss of read depth between taxa, and should be considered for future studies of this nature.

The unique biases and workflows associated with a specific primer pair, or combination of primer pairs, may undermine comparative dietary analyses that use different primer pairs. The ecological conclusions of such comparisons may be weakened given the differential coverage selected primer pairs may afford and how this influences our understanding of a single or multiple focal consumers. Consistent use of primer pairs therefore offers researchers greater power to resolve and analyse differences between focal species, which is increasingly important considering the increasing prevalence of multitaxon studies and multiple studies of the same taxon. Therefore, we ultimately encourage researchers to favour consistency above specificity in primer selection for multitaxon studies, or for comparative analyses between studies.

## 4 ALTERNATIVE METHODS FOR THE EXCLUSION OF PREDATOR DNA IN METABARCODING

Whilst the common methods for dealing with the predator problem are inconsistent in their success and can introduce additional issues, there are other solutions, some of which are yet to be applied to dietary analysis. The solutions discussed above all concern the PCR stage, but the metabarcoding workflow presents other intervention points that warrant consideration for addressing the predator problem (Table 1).

**TABLE 1:** The discussed approaches for overcoming the predator problem in molecular dietary analysis and the problems they present for single‐species and multitaxon studies

Stage	Approach	Result	Single‐species problems	Multitaxon problems
Pre‐PCR	Exclusion of long‐fragment DNA	Removal of long‐fragment DNA prior to PCR, which will probably be predominantly intact/fresh predator DNA, leaving more of the degraded prey DNA	Freshly consumed prey DNA may be removed and the degraded predator DNA (e.g., depleted tissue inherent to guts/faeces) will still remain, although possibly in a lower proportion	If the predators have different feeding modes/physiologies (e.g., fresh fluid feeding vs. coprophagy), or if the sample material differs (e.g., faeces vs. gut contents) this method might inconsistently affect data output
	Bait‐based removal	Specific predator DNA can be extracted prior to PCR, reducing or eliminating its prevalence at the PCR stage	Phylogenetically proximate prey may also be removed. Different baits may be required if using multiple markers (e.g., mitochondrial and ribosomal baits for COI and 16S)	Multiple baits may be necessary to cover each predator, increasing costs, complexity and labour. If using only one bait, some predator DNA will probably persist, depending on taxonomic proximity and bait specificity
PCR	General amplification including the predator	Large amounts of sequencing depth lost to the predator but a diverse range of prey detected otherwise (if at all)	Loss of prey data	Possible differential amplification of the predators and thus inconsistent read depths between predator taxa
	Exclusion primers	Prevention of amplification of specific predator DNA	Possible exclusion of taxonomic compartments of prey	If using different primers for each predator, inconsistent effects on prey exclusion. If using the same primers, inconsistent read depth between predators
	Blocking probes	Prevention of amplification of specific predator DNA	Possible exclusion of some prey taxa and inconsistent success	If using different probes for each predator, inconsistent effects on prey exclusion. If using the same probes, inconsistent read depth between predators
	Multiprimer approach with general and exclusion primers	Breadth of prey taxa achieved via general primers, and depth of sequencing achieved by exclusion primers	The loss of reads by general primers and taxonomic bias of exclusion primers, but no problems unique to their combination	The differential bias between different predator taxa persists, but not uniquely to this combined approach
Post‐PCR	DASH	Post‐PCR exclusion of predator DNA from sequencing	If the predator DNA comprises a large contingent of the PCR product, the yield at the sequencing stage may be unpredictably low	If using different sgRNAs for each predator, inconsistent effects on prey exclusion. If using the same sgRNA, inconsistent read depth between predators
PCR‐free	Bait‐based isolation and enrichment	Specific prey mitochondrial DNA can be extracted, isolated and enriched from extracts, whilst neglecting the predator DNA, or using different baits to remove this, as above	Highly degraded DNA may present challenges. Exclusion of predator DNA, if not using additional baits to remove it, may introduce taxonomic biases against prey or may be ineffective	Exclusion of multiple predators, especially if phylogenetically distant, may prove difficult without introducing bias, unless using predator‐specific baits to remove each predator prior to enrichment
	Metagenomics (e.g., shotgun sequencing)	Eliminates the bias of PCR	Expensive and requires sufficient DNA yield across samples. The prevalence of predator DNA may still be problematically high, unless paired with baiting or a similarly selective method	No specific multitaxon issues immediately evident

Krehenwinkel, Kennedy, et al. (2017) suggested that removal of longer oligonucleotides via size selection techniques such as solid phase reversible immobilization (SPRI) beads or pulsed‐field electrophoresis equipment such as the Blue Pippin (Sage Science) could be an effective means for reducing the prevalence of predator DNA in metabarcoding. This is based on the notion that prey DNA, being digested, will typically be fragmented into smaller molecules than the fresh intact DNA of a predator (Sint & Raso, 2011; Waldner et al., 2013). Since all gut and faecal contents will include some degraded predator DNA, this will not provide a perfect removal of the problem but may significantly improve data output. No published studies have yet replicated this approach though. Importantly, if effective, this might enact bias against recently consumed prey, although this is unlikely to be problematic for externally digesting predators such as arachnids, the context in which the method was initially presented. The efficacy of techniques such as this should be considered carefully for multitaxon studies, especially if the feeding mode (e.g., fluid feeding vs. coprophagous, digestive tract size/intensity) or sample type (e.g., faeces vs. gut contents) of the predators differs, which may affect gut DNA half‐life or predator DNA degradation and therefore affect the difference in fragment size between predator and prey DNA (Paula et al., 2015; von Berg et al., 2008; Waldner et al., 2013).

It is also possible to restrict the impact of predator amplicons post‐PCR by interfering with their interaction with the sequencer. Depletion of abundant sequences by hybridisation (DASH) is a method proposed for molecular studies that are burdened with high proportions of nontarget DNA (Gu et al., 2016; Ramani & Shendure, 2016). DASH involves hybridizing the unwanted DNA with recombinant Cas9 endonuclease via specific single guide RNAs (sgRNAs) and cleaving one of the indexes prior to final amplification of the library, effectively preventing amplification of these sequences and their downstream sequencing. The specificity of this approach relies on purpose‐designed sgRNAs, but these must be proximate to an existing protospacer adjacent motif (PAM) site immediately downstream from the cut site. This imposes a restriction on where amplicons can be cut, although different Cas endonucleases can be used, each with its own specific PAM site sequence requirements (e.g., the most common Cas9, *Sp*Cas9, requires NGG; Hu et al., 2018). This method appears promising for dietary analyses, but this late‐stage intervention (i.e., post‐PCR) could prove problematic in instances of high predator amplicon prevalence since the remaining yield could be unpredictably low. The specificity of sgRNAs for phylogenetically proximate predators and prey also requires some empirical validation in this context, particularly for application to multitaxon studies.

Target enrichment methods such as “baiting” (sequence‐specific hybridisation capture) of specific organellar genomes or gene regions has demonstrated great success with various environmental and ecological samples (Aylward et al., 2018; Seeber et al., 2019), even degraded and fragmented DNA (Kollias et al., 2015), but has yet to be applied in a dietary context. Specific baits can be produced for the isolation, or removal, of DNA of a given taxon, which could theoretically be applied to the removal of predator DNA, for example its mitochondrial genome, to limit or even eliminate its prevalence in downstream PCR. This would be achieved by designing baits to complement predator DNA, hybridizing the baits to predator DNA in samples, and removing the supernatant, leaving behind the baits hybridized to a large proportion of the predator DNA. The likelihood of off‐target effects of this are yet to be fully explored, but some removal of phylogenetically proximate prey DNA could be expected. For multitaxon studies, multiple baits would possibly have to be generated, each with their own possible nontarget effects, but this method could theoretically present a viable advance for dietary studies faced with the predator problem.

This baiting approach would also be a viable means for removing predator DNA prior to PCR‐free metagenomic analysis, which avoids the biases associated with PCR (Paula et al., 2016). PCR‐free metagenomics theoretically improves the taxonomic breadth of consumed species identified, semi‐quantification of consumption events and the potential for lateral data collection (e.g., parasites, microbiome, endosymbionts; Chua et al., 2021; Paula et al., 2016; Srivathsan et al., 2014, 2016), but at a greater overall cost given the need for increased sequencing depth and the higher incidence of nontarget reads. This constraint could be somewhat alleviated for dietary applications by adoption of a bait‐based approach to removing predator DNA, but this importantly would only remove specific targets, leaving nontarget DNA that may consume sequencing depth. If sufficiently general baits could be designed, this approach could, however, first be used to retain only DNA from specific organelles, which could then be enriched irrespective of removal of the predator. Through isothermal amplification, such as rolling circle amplification, often‐circular whole organelle genomes could be amplified and concatemeric branches accurately sequenced using accessible long‐read sequencing platforms such as nanopore sequencing (Baloğlu et al., 2021). Such approaches are yet to be demonstrated in empirical dietary research but could present a natural progression away from DNA metabarcoding approaches for increased taxonomic resolution and breadth, and reduced bias. A substantial body of research must first be completed to validate these approaches, alongside increased accessibility to long‐read and increasingly deep sequencing platforms.

## 5 CONCLUSIONS

The predator problem is a pervasive and widespread issue in molecular dietary analyses, with profound and unaddressed implications, particularly for increasingly common multitaxon studies such as those constructing complex ecological networks (Clare et al., 2018; Hemprich‐Bennett et al., 2021; Ingala et al., 2021; Mata et al., 2021). The methods used to overcome the problem are variably effective and present significant trade‐offs, particularly regarding bias and the amount of data output. We suggest that dietary studies use multiple primer pairs with an emphasis on normalizing bias and prey read depth across studies. The less common methods presented as possible solutions to the predator problem require additional empirical exploration to ascertain their efficacy before widespread adoption by the dietary metabarcoding community.

The predator problem is laterally relevant to other contexts such as filter feeding (Zamora‐Terol et al., 2020) and parasitism (Miller et al., 2021). Metabarcoding studies should consider the relevance of this problem before embarking on empirical work. Regardless of the solution selected, there are several key steps that can be taken to understand the extent to which the predator problem is likely to affect each study. Empirical pilot studies can be invaluable in identifying downstream problems for metabarcoding studies (Browett et al., 2021), although these can inflate overall costs. PCR primers must also be tested prior to working with samples. Primers can be tested in silico (i.e., computationally through simulated PCR reactions) to evaluate the likelihood of amplifying the target species prior to investing in sequencing or in vitro (i.e., laboratory‐based; Clarke et al., 2014; Elbrecht & Leese, 2016; Ficetola et al., 2010; MacDonald & Sarre, 2016). Results from in vitro testing, which can differ substantially from in silico results, can then be used to confirm the range of taxa amplified by the primer pair. Given that each PCR primer pair exhibits a distinct taxonomic bias which profoundly impacts data output (Alberdi et al., 2018; Taberlet et al., 2018), selection of primers can be among the most crucial decisions in any metabarcoding workflow (Piñol et al., 2018), not least to avoid implications of the predator problem. Arguably most crucially though, is the transparent and clear reporting of the extent to which the predator problem impacts each study. This should not be viewed as a detraction from the research itself, but rather a hallmark of open science in molecular dietary analysis. By making this information accessible, future studies, particularly those in similar systems, can refine their approach to mitigate the potentially large impacts of the predator problem.

## CONFLICTS OF INTEREST

There are no conflicts of interest to declare.

## Supporting information


Appendix S1

## Data Availability

The data and R scripts used for Figure 4 have been made available via Zenodo: https://doi.org/10.5281/zenodo.6501587 (Cuff, Kitson, et al., 2022). Raw sequencing data, processed data and R scripts for Figure 3 were published by Cuff, Tercel, et al. (2022) and are separately available via Zenodo: https://doi.org/10.5281/zenodo.4708418 (Cuff, 2021).
