# Supplementary material for: The predator problem and PCR primers in molecular dietary analysis: Swamped or silenced; depth or breadth?
Source: Mol Ecol Resour. 2022 Sep 10;23(1):41–51. doi: 10.1111/1755-0998.13705 (PMC10087656; doi:10.1111/1755-0998.13705)
Supplement: Supplementary file 1 — Appendix S1 [file MEN-23-41-s001.docx]

**Supplemental Information for:**

**The predator problem and PCR primers in molecular dietary analysis: swamped or silenced; depth or breadth?**

Jordan P. Cuff, James J.N. Kitson, David Hemprich-Bennett, Maximillian P.T.G. Tercel,

Samuel S. Browett, Darren M. Evans

**Table of Contents:**

| **Supplementary Information** | Page 1 |
| --- | --- |
| **Supplementary Tables** | Page 2 |

**Supplementary Information 1**

Data were collected and processed according to Cuff, Tercel, et al., (2022) up to the point of aggregating the two primer pair datasets. Binary matrices for prey detections were combined for the two primer pairs, but each sample represented separately for each primer pair (i.e., not aggregated by sample). Instances where taxa were only identified to genus (or lower, e.g., family) level by only one of the primer pairs resulted in aggregation for the other primer pair at that taxonomic level, except for species within those groups that were reliably identified to species level by both primers. Samples for which only one primer pair generated prey data were removed. The non-metric multidimensional scaling spider plot was created using ‘metaMDS’ with a Jaccard distance matrix and 999 tries in the ‘vegan’ package (Oksanen et al., 2016). Outliers that obscured the overall patterns were removed, the final plot having a stress of 0.061. Point colours were assigned using the ‘set1’ palette of the ‘RColorBrewer’ package (Neuwirth, 2014) and the final plot created using ‘ggplot2’ (Wickham, 2016).

**Supplementary Tables**

Table S1: The percentage of reads attributed to predator and prey across five spider genera and two primer pairs.

| **Primer pair** | **Reads** | ***Bathyphantes*** | ***Erigone*** | ***Microlinyphia*** | ***Pardosa*** | ***Tenuiphantes*** |
| --- | --- | --- | --- | --- | --- | --- |
| **General** | **Predator** | 99.74 ± 0.78 | 99.05 ± 2.11 | 99.63 ± 0.74 | 98.92 ± 1.71 | 93.82 ± 12.34 |
|  | **Prey** | 0.26 ± 0.78 | 0.95 ± 2.11 | 0.37 ± 0.74 | 1.08 ± 1.71 | 6.18 ± 12.34 |
| **Exclusion** | **Predator** | 75.00 ± 34.48 | 0.00 ± 0.00 | 26.62 ± 31.84 | 99.14 ± 4.60 | 34.84 ± 36.54 |
|  | **Prey** | 25.00 ± 34.48 | 100 ± 0 | 73.38 ± 31.84 | 0.86 ± 4.60 | 65.16 ± 36.54 |
